# Meta-analysis of overall incidence and risk of ALK inhibitors-induced liver toxicities in advanced non-small-cell lung cancer

**DOI:** 10.1097/MD.0000000000013726

**Published:** 2019-01-04

**Authors:** Jingjie Li, Zhi Yuan, Qun Wang, Weijie Fan, Guoping Zhang

**Affiliations:** Department of Respiratory Medicine, Renmin Hospital, Fenghua District, Ningbo City, Zhejiang Province, China.

**Keywords:** ALK inhibitors, liver toxicity, meta-analysis, non-small-cell lung cancer

## Abstract

**Aim:**

Activation of the anaplastic lymphoma kinase (*ALK*) gene has been found in several human cancers, including non-small-cell lung cancer (NSCLC). Currently, novel drugs targeting *ALK* gene have been extensively investigated in NSCLC. However, concerns about ALK inhibitors-induced liver toxicities have been increasing.

**Materials and Methods:**

Eligible prospective clinical studies have been searched in several databases. Primary outcomes of interest were incidence rates of liver toxicities, relative risks (RRs), and 95% confidence intervals (CIs).

**Results:**

Data from 2418 patients (1873 in the experimental arm; 545 in the control arm) were included. The incidences of all-grade alanine transaminase (ALT) and aspartate aminotransferase (AST) elevation were 26.0% (95% CI: 17.4%–37%), and 23.2% (95% CI, 16.7%–31.4%), respectively. The incidences of high-grade ALT and AST elevation were 8.4% (95% CI, 5.1%–13.4% and 7.0% (95% CI: 5.4%–9.0%), respectively. Sub-group analysis according to the ALK inhibitors found that pooled incidence of liver toxicities associated with ceritinib was higher than that of crizotinib and alectinib. In comparison with chemotherapy, ALK inhibitors significantly increased the all-grade and high-grade ALT elevation (RR 2.37, 95% CI, 1.97–2.86; *P < *.001; RR 7.34, 95% CI, 3.95–13.63; *P < *.001) and AST elevation (RR 3.27, 95% CI, 2.47–4.34; *P < *.001; RR 11.54, 95% CI, 4.33–30.7; *P < *.001), respectively. No publication bias was detected for RR of ALT and AST.

**Conclusions:**

The findings of the present study offer substantial evidence that ALK inhibitors treatment in advanced NSCLC significantly increases the risk of developing all-grade and high-grade liver toxicities in comparison with controls. Clinicians should recognize liver toxicities promptly as early interventions may alleviate future complications.

## Abstract 2

Supplemental Digital Content is available in the text

## 1 Introduction

Lung cancer is a leading cause of cancer related mortalities around the world.^[1]^ Approximately 80%–85% of lung cancer cases could be diagnosed as non-small-cell lung cancer (NSCLC).^[2]^ Unfortunately, the prognosis of NSCLC remains poor, with a 5-year survival rate of 16% and more than 40%–50% is presented with advanced disease. For patients with advanced NSCLC, platinum-based chemotherapy remains the standard of care, which has a response rate of approximately 30%, and the response usually lasts only 4 to 5 months. During the past decade, more advances in the understanding of the pathogenesis of NSCLC have led to the introduction of a variety of biological agents into clinical practice.^[3–5]^ The epidermal growth factor receptor (EGFR) activating mutations are the first oncogenic drivers to be discovered in advanced NSCLC.^[6]^ Multiple prospective clinical randomized trials have clearly shown that EGFR-tyrosine kinase inhibitors (TKIs), including erlotinib, gefitinib or afatinib, are superior than that of conventional chemotherapy.^[7–10]^ NSCLC harboring an anaplastic lymphoma kinase (ALK) -rearrangement represent the second oncogene addiction, which accounts for approximately 5% of advanced adenocarcinoma.^[11,12]^ ALK fusion proteins promote tumor cell growth and survival through the aberrant activation of intracellular signaling. Specific ALK- TKIs have been developed during the past decade. The first approved ALK inhibitor, crizotinib, significantly improved progression-free survival compared with chemotherapy in advanced NSCLC with ALK-positive fusion. Another selective ALK inhibitor, Alectinib, also demonstrated improved survival and high central nervous system (CNS) penetration in advanced NSCLC with ALK-positive fusion.^[13,14]^

Generally, although ALK inhibitors are well tolerated, a unique toxicities profiles associated with these drugs have been observed, which are different from traditional cytotoxic anticancer therapies.^[15–17]^ For instance, previous studies have shown an increased risk of all-grade stomatitis, skin rash, diarrhea, nausea, and elevated transaminases. However, there has been a substantial variation in the incidence of hepatic adverse events (AEs) among clinical trials, with some studies reporting increased risk while the others do not. Additionally, there has been no systematic attempt to synthesize these data and the overall risk of hepatic toxicities induced by ALK inhibitors has yet to be defined.^[18]^ In addition, current understanding of liver toxicity risk based on individual trial is limited due to small sample size and patient selection in these clinical studies. Therefore, we conducted a systematic review of published phase II and III clinical trials, and combined relevant studies for a meta-analysis to evaluate the overall risk of liver toxicity during the administration of ALK inhibitors.

## 2 Materials and methods

### 2.1 Data sources

We conducted an independent review of Pubmed, Embase, and the Cochrane Library electronic databases from Jan 2000 to Jan 2018 by using the following key-words: “ALK-TKIs”, “ALK inhibitors”, “crizotinib”, “ceritinib”, “alectinib”, “NSCLC”, and “liver toxicities”. The search was limited to human, cancer, and randomized clinical trials published in English. We manually searched abstracts and presentations containing the same search term ‘ALK inhibitors’ from the American Society of Clinical Oncology (ASCO) conferences held between January 2006 and January 2018 to search for relevant trials. An independent search of the Google Scholar and Cochrane electronic databases was also performed to ensure that no additional clinical trials had been overlooked. In cases of duplicate publications, only the most complete, recent and updated report of the clinical trial was included. Finally, the most updated package insert from crizotinib, alectinib, and ceritinib was reviewed to identify relevant information. Trials were selected and systemically reviewed in accordance with the Preferred Reporting Items for Systematic Reviews and Meta-Analyses (PRISMA) statement.

### 2.2 Study Selection

Clinical trials that met the following criteria were included:

1. prospective phase II or III trials involving NSCLC patients;
2. patients assigned to treatment with ALK inhibitors daily;
3. events or event rate and sample size available for all-grade and high-grade alanine aminotransferase (ALT) and the increase of aspartate aminotransferase (AST);
4. For incidence analysis and relative risk (RR) analysis, we included trials that randomly assigned participants to either ALK inhibitors versus placebo or control drug in addition to the same treatment.

### 2.3 Exclusion criteria included

1. Phase I trial because of the different drug dosages as well as the small number of patients in these trials.
2. Meeting abstracts without subsequent full-text publication were also excluded. Independent reviewers screened reports that included the key term by their titles and abstracts for relevance. Then, full texts of the relevant articles were retrieved to assess eligibility. The references of relevant reports were also reviewed manually.

### 2.4 Data extraction and clinical end point

Data abstraction was conducted independently by 2 investigators, and any discrepancy between the reviewers was resolved by consensus. For each study, the following information was extracted: first author's name, year of publication, trial phase, number of enrolled subjects, treatment arms, number of patients in treatment and control groups, median age, median progression-free survival, and adverse outcomes of interest (liver toxicities).

Three variables were separately considered such as expression of hepatotoxicity: the increase of ALT and AST. For each variable, we consider the increase of all grades and grade 3 to 4 as the main outcomes and the analysis was conducted in order to find a significant difference between the two arms. AEs were defined as per version 3.0 of the National Cancer Institute's Common Terminology Criteria for AEs criteria because of its use in the selected trials (NCI-CTC, version 2 or 3; http://ctep.cancer.gov). In the event a study reported high-grade but not low-grade liver toxicities, no assumption of all-grade incidence was made.

### 2.5 Statistical Analysis

For the calculation of incidence, the number of patients with liver toxicities in ALK inhibitors group and the total number of patients receiving ALK inhibitors were extracted from the selected clinical trials; the proportion of patients with infections and 95% confidence interval (CI) were derived for each study. To calculate RR, patients assigned to ALK inhibitors were compared only with those assigned to control treatment in the same trial. For 1 study that reported 0 events in the treatment or control arm, we applied the classic half-integer correction to calculate the RR and variance.^[19]^ Between-study heterogeneity was estimated using the *χ*^2^-based Q statistic.^[20]^ Heterogeneity was considered statistically significant when *P*_heterogeneity_ <.05. If heterogeneity existed, the pooled estimate calculated based on the random-effects model was reported using the DerSimonian et al method.^[21]^ In the absence of heterogeneity, the pooled estimate calculated based on the fixed-effects model was reported using inverse variance method. A statistical test with a *P*-value less than .05 was considered significant. The presence of publication bias was evaluated by using the Begg and Egger tests.^[22]^ The Jadad scale was used to assess the quality of included trials based on the reporting of the studies’ methods and results.^[23]^ We did all statistical analyses with open Meta-Analyst software version 4.16.12 (Tufts University, URL http://tuftscaes.org/open_meta/) and SPSS18.0 software (SPSS Inc., Chicago, IL, United States).

## 3 Results

### 3.1 Search results

**Figure 1:**
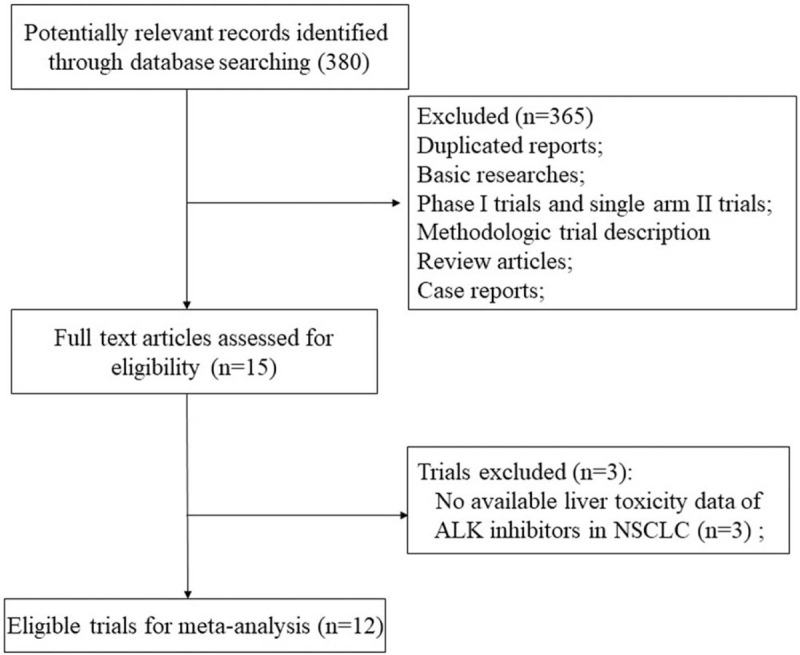
Flow chart of trial selection process in the meta-analysis.

Our search strategy yielded 380 potentially relevant citations on ALK inhibitors from PubMed/Medline, Cochrane registry and ASCO meeting library. The reasons for study exclusion are shown in Figure 1. Thus, a total of 12 clinical trials were considered eligible for the meta-analysis, including 5 Phase III trials^[24–28]^ and 7 Phase II trials.^[29–35]^

### 3.2 Population characteristics

A total of 2418 patients were included for analysis. The characteristics of patients and studies were listed in Table 1. The baseline Eastern Cooperative Oncology Group performance status for the majority of patients was between 0, 1 and 2. According to the inclusion criteria of each trial, patients were required to have adequate hepatic, renal and hematological function. All of the five randomized controlled trials were open-label controlled trials, thus had Jadad score of 3.

**Table 1:**
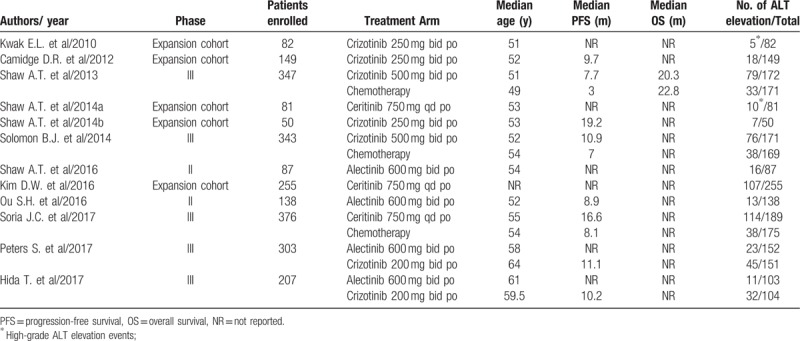
baseline characteristics of 12 included trials.

### 3.3 Incidence and relative risk of ALT increase

**Figure 2:**
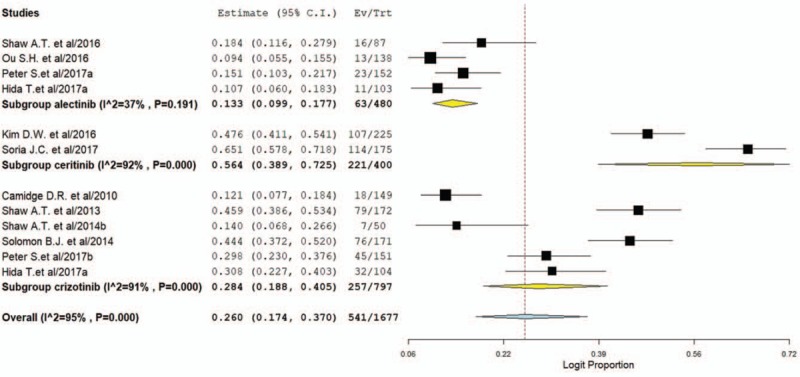
Pooled incidence of all-grade ALT elevation in NSCLC patients treated with ALK inhibitors. ALK = anaplastic lymphoma kinase, ALT = alanine transaminase, NSCLC = non-small-cell lung cancer.

For incidence of any grade of ALT increase, a total of 1677 patients were included in the analysis: the increase of the ALT was reported in 541 out of 1677 ALK inhibitors treated patients with an incidence of 26.0% (95% CI: 17.4%–37%, Fig. 2). Sub-group analysis according to the ALK inhibitors showed that the incidence of ALT associated with ceritinib (56.4%, 95% CI: 38.9%–72.5%) was significantly higher than that of alectinib (13.3%, 95% CI: 9.9%–17.7%) and crizotinib (28.4%, 95% CI: 18.8%–40.5%). The RR (fixed effect) to develop any grade of ALT increase was 2.37 (95% CI, 1.97–2.86; *P < *.001) in patients treated with ALK inhibitors compared to chemotherapy (*P*=.37; *I*^2^ = 0%, supplemental Fig. 1).

**Figure 3:**
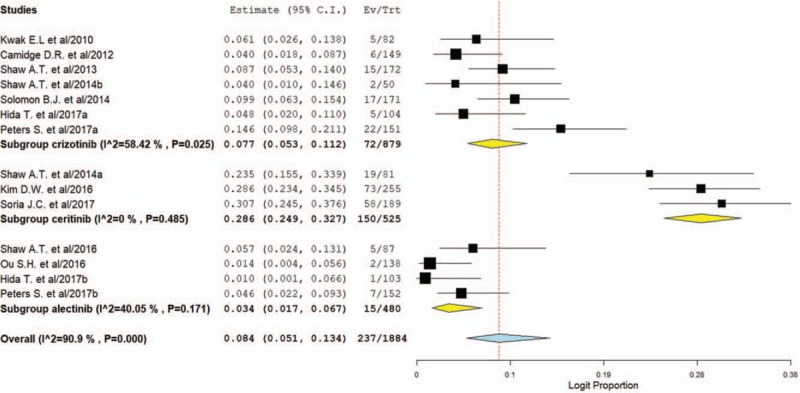
Pooled incidence of high-grade ALT elevation in NSCLC patients treated with ALK inhibitors. ALK = anaplastic lymphoma kinase, ALT = alanine transaminase, NSCLC = non-small-cell lung cancer.

The grade 3 to 4 of the ALT increase was evaluable in 1884 patients and the incidence of high grade of ALT increase was 8.4% (95% CI, 5.1%–13.4%, Fig. 3) for ALK inhibitors. The RR to develop grade 3 to 4 of ALT increase was 7.34 (95% CI, 3.95–13.63; *P < *.001) in patients treated with ALK inhibitors compared to chemotherapy (supplemental Fig. 2). No significant heterogeneity was observed in the RR analysis for grade 3 to 4 (*P* = .27; *I*^2^ = 23.4%).

### 3.4 Incidence and relative risk of AST increase.

**Figure 4:**
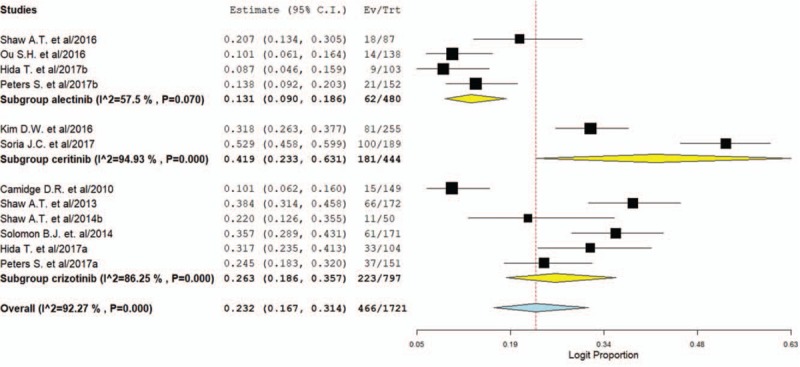
Pooled incidence of all-grade AST elevation in NSCLC patients treated with ALK inhibitors. ALK = anaplastic lymphoma kinase, AST = aspartate aminotransferase, NSCLC = non-small-cell lung cancer.

For incidence of any grade of AST increase, a total of 1721 patients were included in the analysis: the increase of the AST was reported in 466 out of 1721 ALK inhibitors treated patients with an incidence of 23.2% (95% CI, 16.7%–31.4%, Fig. 4). Sub-group analysis according to the ALT inhibitors showed that the incidence of AST elevation associated with ceritinib (41.9%, 95% CI: 23.3%–63.1%) was higher than that of alectinib (13.1%, 95% CI: 9.0%–18.6%) and crizotinib (26.3%, 95% CI: 18.6%–35.7%). The RR (fixed effect) to develop any grade of AST increase was 3.27 (95% CI, 2.47–4.34; *P < *.001, supplemental Fig. 3) in patients treated with ALK inhibitors compared to controls.

**Figure 5:**
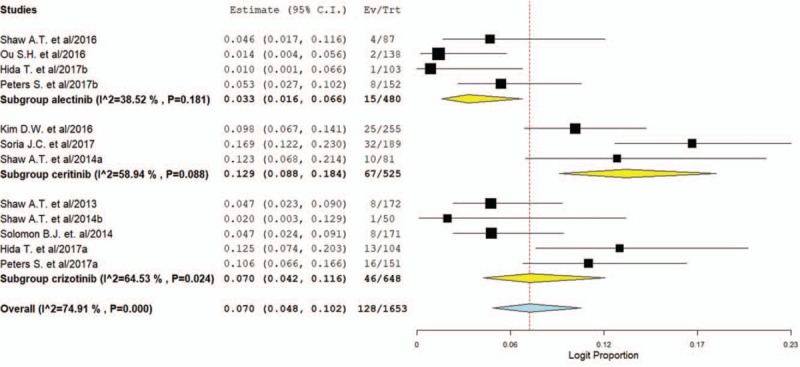
Pooled incidence of high-grade AST elevation in NSCLC patients treated with ALK inhibitors. ALK = anaplastic lymphoma kinase, AST = aspartate aminotransferase, NSCLC = non-small-cell lung cancer.

The grade 3 to 4 of the AST increase was evaluable in 1653 patients and the incidence of high grade of AST increase was 7.0% (95% CI, 4.8%–10.2%, Fig. 5) for ALK inhibitors. The RR to develop grade 3 to 4 of the AST increase (fixed effect) was 11.54 (95% CI, 4.33–30.7; *P < *.001, supplemental Fig. 4) in patients treated with ALK inhibitors compared to controls. No significant heterogeneity was observed with fixed model in the analysis for all grades (*P* = .12; *I*^2^ = 52.6%) and grade 3 to 4 (*p* = 0.89; *I*^2^ = 0%) of AST increase.

### 3.5 Publication bias

No significant publication biases were detected for all grades of hepatic toxicities: *P*-values from Begg and Egger test were 0.54 and 0.62 for ALT increase, 0.50 and 0.56 for AST increase, respectively (supplemental Fig. 5). Similarly, no significant publication biases were detected for high grades of ALT and AST: *P* values from Begg and Egger test were 0.60 and 0.69, 0.60 and 0.81, respectively.

## 4 Discussion

The present study is the most comprehensive meta-analysis to specially assess the incidence and risk of liver toxicities with administration of ALK inhibitors in NSCLC. Our result has demonstrated that ALK inhibitors are associated with a significantly increased risk of liver toxicity based on the meta-analysis of 2418 patients (1873 in the experimental arm; 545 in the control arm) from 12 clinical trials. The incidences of all-grade ALT and AST elevation are 26.0% (95% CI: 17.4%–37%), and 23.2% (95% CI, 16.7%–31.4%), respectively. The incidences of high-grade ALT and AST elevation are 8.4% (95% CI, 5.1%–13.4% and 7.0% (95% CI: 5.4%–9.0%), respectively. Sub-group analysis according to the ALK inhibitors finds that pooled incidence of liver toxicities associated with ceritinib is higher than that of crizotinib and alectinib. In comparison with chemotherapy, ALK inhibitors significantly increase all-grade and high-grade ALT elevation (RR 2.37 and RR 7.34) and AST elevation (RR 3.27 and RR 11.54), respectively. Based on our findings, physicians and patients could fully understand the risk of drug-induced liver injury (DILI) with ALK inhibitors in NSCLC patients. Due to the approval of its application in ALK-positive NSCLC patients, these drugs will be increasingly used in routine cancer therapy as well as clinical trials. Awareness of such risks and close monitoring could permit early appropriate intervention to reduce morbidity and mortality associated with liver damage.

Drug-induced liver injury remains the most common AEs resulting in product withdrawals and study terminations. Several theories regarding its pathogenesis have been postulated including immune-mediated toxicity, mitochondrial dysfunction, variations in host metabolic response, or less commonly, direct toxicity to hepatocytes. However, the specific mechanism underlying TKI-related hepatic toxicity is still not well clarified, further studies are recommended to address these issues.

Current recommendations for the monitoring and management of ALT inhibitors induced liver toxicity are mostly based on the experiences from clinical trials. Pre-treatment laboratory workup should include baseline liver function tests followed by transaminase monitoring every 2 weeks during the first 2 months, then monthly and as clinically indicated, with more frequent repeat testing for increased liver transaminases, alkaline phosphatase, or total bilirubin in patients who develop transaminase elevations.^[18]^ The manufacturer has recommended a dose adjustment for baseline moderate hepatic impairment. It is currently contraindicated in patients with severe hepatic impairment. The majority of susceptible patients will experience liver enzyme elevations in the first few months of drug exposure and return to baseline levels upon treatment interruption. In most studies used in our analysis, dose interruptions, adjustments, or discontinuations were made in response to raised transaminase levels. For grade 3 or higher aminotransferase elevations, ALK inhibitor was typically held until return to pretreatment levels. ALK inhibitor was then resumed at reduced dose.

There are several limitations need to be mentioned. First and most importantly, the application of formal meta-analytic methods to single arm studies has been controversial. One of the most important reasons for this is that the designs and populations of the studies are diverse, and that these differences may influence the pooled estimates. Second, elevation of ALT, AST, and bilirubin represents liver injury but these tests do not have great sensitivity or specificity. Third, patients in trials have adequate organ and hematological function, which may not be the case in common oncology practice. All of these might cause potential selection bias. Finally, this is a meta-analysis of published data, and lack of individual patient data prevents us from adjusting the treatment effect according to previous treatment and patient variables.

## 5 Conclusion

In conclusion, the findings of the present study offer substantial evidence that ALK inhibitors treatment in advanced NSCLC significantly increases the risk of developing all-grade and high-grade liver toxicities in comparison with controls. Clinicians should recognize liver toxicities promptly as early interventions may alleviate future complications. In addition, more trials are still needed to investigate the potential predictive factors in order to avoid toxicity and premature drug discontinuation.

## Author contributions

**Conceptualization:** Jingjie Li, Guoping Zhang.

**Data curation:** Zhi Yuan.

**Investigation:** Zhi Yuan, Weijie Fan, and Guoping Zhang.

**Methodology:** Weijie Fan.

**Project administration:** Qun Wang, Weijie Fan, and Guoping Zhang.

**Resources:** Zhi Yuan and Qun Wang.

**Software:** Zhi Yuan, Qun Wang, and Guoping Zhang.

**Supervision:** Jingjie Li.

**Validation:** Jingjie Li.

**Visualization:** Qun Wang.

**Writing – original draft:** Jingjie Li.

**Writing – review & editing:** Jingjie Li.

## Supplementary Material

Supplemental Digital Content
