# Supplementary material for: Meta-analysis of overall incidence and risk of ALK inhibitors-induced liver toxicities in advanced non-small-cell lung cancer
Source: Medicine (Baltimore). 2019 Jan 4;98(1):e13726. doi: 10.1097/MD.0000000000013726 (PMC6344205; doi:10.1097/MD.0000000000013726)

Studies	Estimate (95% C.I.)	Ev/Trt	Ev/Ctrl
Shaw A.T. et al/2013	2.380 (1.683, 3.366)	79/172	33/171
Solomon B.J. et al/2014	1.977 (1.426, 2.739)	76/171	38/169
Soria J.C. et al/2017	2.778 (2.049, 3.765)	114/189	38/175
**Overall (I^2=0 % , P=0.327)**	**2.374 (1.968, 2.863)**	**269/532**	**109/515**

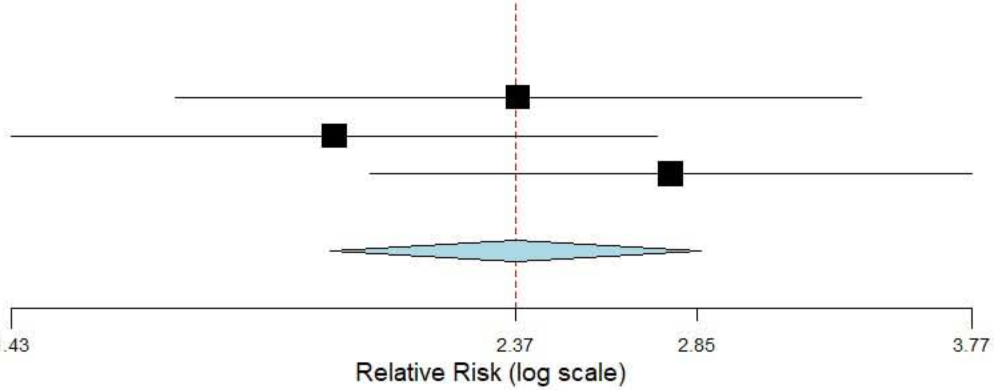

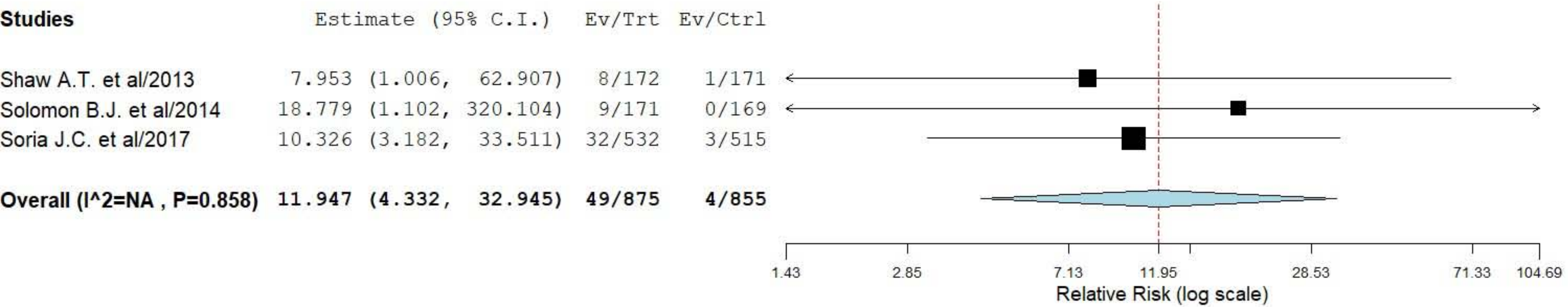
Studies
Estimate (95% C.I.)
Ev/Trt
Ev/Ctrl
Shaw A.T. et al/2013 7.953 (1.006, 62.907) 8/172 1/171
Solomon B.J. et al/2014 18.779 (1.102, 320.104) 9/171 0/169
Soria J.C. et al/2017 10.326 (3.182, 33.511) 32/532 3/515
Overall (I^2=NA , P=0.858) 11.947 (4.332, 32.945) 49/875 4/855
1.43
2.85
7.13
11.95
28.53
71.33
104.69
Relative Risk (log scale)

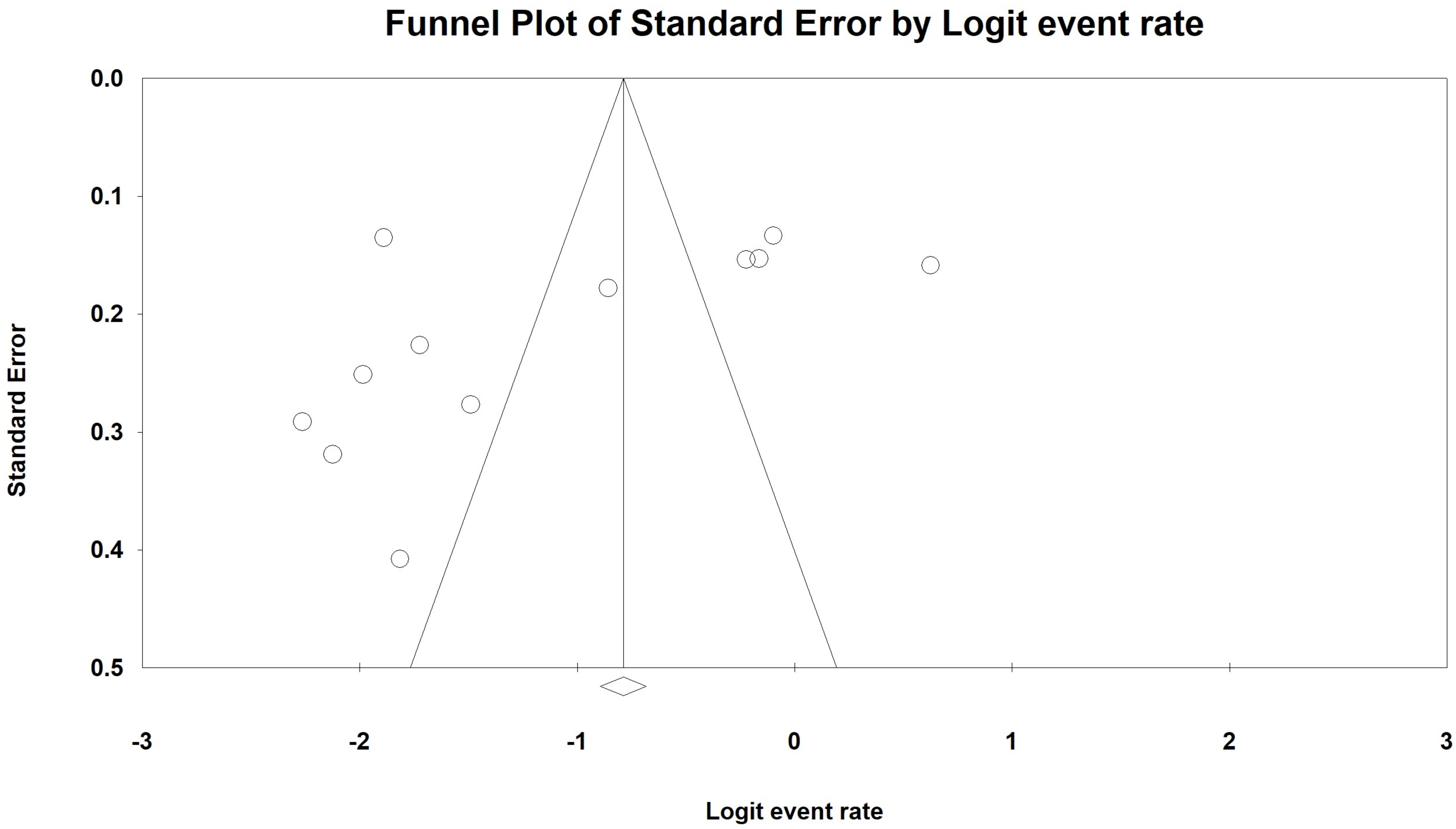

Funnel Plot of Standard Error by Logit event rate
0.0
0.1
0.2
0.3
0.4
0.5
Standard Error
-3
-2
-1
0
1
2
3
Logit event rate

Studies	Estimate (95% C.I.)	Ev/Trt	Ev/Ctrl
Shaw A.T. et al/2013	2.343 (1.590, 3.455)	66/172	28/171
Solomon B.J. et al/2014	1.827 (1.267, 2.635)	61/171	33/169
Soria J.C. et al/2017	2.313 (1.881, 2.844)	227/532	95/515
**Overall (I^2=NA , P=0.519)**	**2.216 (1.883, 2.609)**	**354/875**	**156/855**

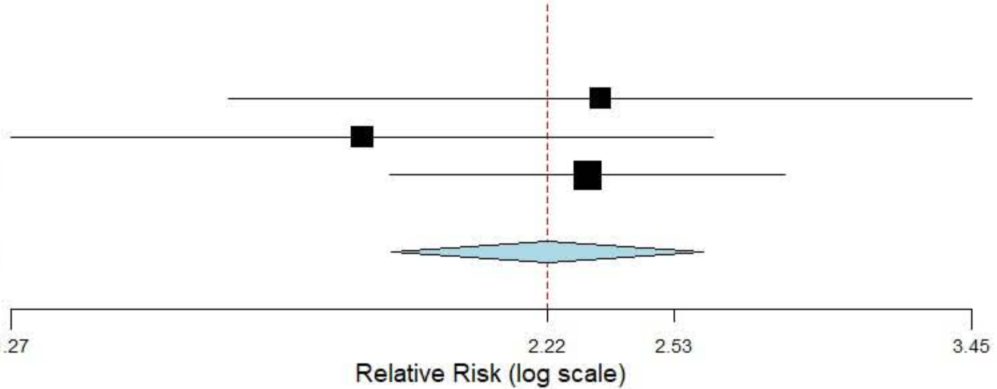

Studies	Estimate (95% C.I.)	Ev/Trt	Ev/Ctrl
Shaw A.T. et al/2013	7.456 (1.731, 32.112)	15/172	2/171
Solomon B.J. et al/2014	4.200 (1.443, 12.224)	17/171	4/169
Soria J.C. et al/2017	10.741 (4.410, 26.157)	58/189	5/175
**Overall (I^2=0 % , P=0.416)**	**7.337 (3.950, 13.628)**	**90/532**	**11/515**

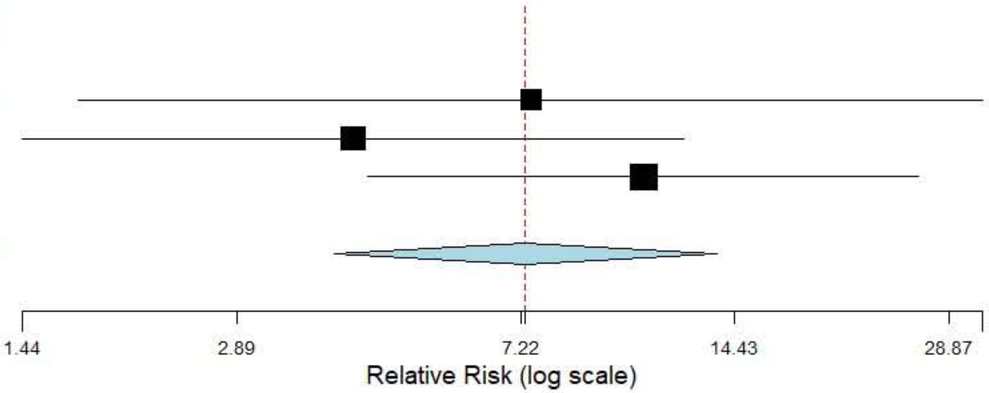

Supplement: Supplemental Digital Content [file medi-98-e13726-s001.pdf]
